# Supplementary material for: A cancer vaccine-mediated postoperative immunotherapy for recurrent and metastatic tumors
Source: Nat Commun. 2018 Apr 18;9:1532. doi: 10.1038/s41467-018-03915-4 (PMC5906566; doi:10.1038/s41467-018-03915-4)
Supplement: Supplementary file 1 — Supplementary Information [file 41467_2018_3915_MOESM1_ESM.pdf]

Supplementary Information

Wang et al. A cancer vaccine-mediated postoperative immunotherapy for recurrent and metastatic tumors.

Table of Contents

Supplementary Figure 1.	Page 3
Supplementary Figure 2.	Page 4
Supplementary Figure 3.	Page 5
Supplementary Figure 4.	Page 6
Supplementary Figure 5.	Page 7
Supplementary Figure 6.	Page 8
Supplementary Figure 7.	Page 9
Supplementary Figure 8.	Page 10
Supplementary Figure 9.	Page 11
Supplementary Figure 10.	Page 12
Supplementary Figure 11.	Page 13
Supplementary Figure 12.	Page 14
Supplementary Figure 13.	Page 15
Supplementary Figure 14.	Page 16
Supplementary Figure 15.	Page 17
Supplementary Figure 16.	Page 18
Supplementary Figure 17.	Page 19
Supplementary Figure 18.	Page 20
Supplementary Figure 19.	Page 21
Supplementary Figure 20.	Page 22
Supplementary Figure 21.	Page 23
Supplementary Figure 22.	Page 24

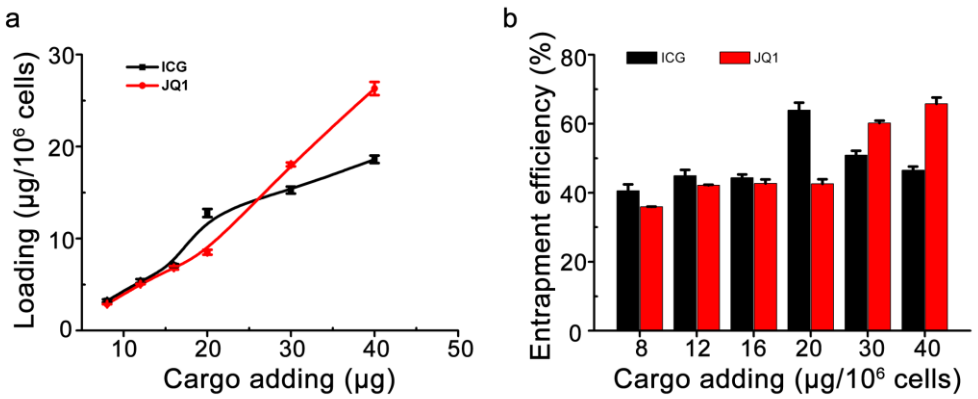

Supplementary Figure 1. Drug loading and encapsulation efficiency of inactivated 4T1 tumor cells. (a) ICG and JQ1 loading ratios, and (b) encapsulation efficiency in 4T1 cells.

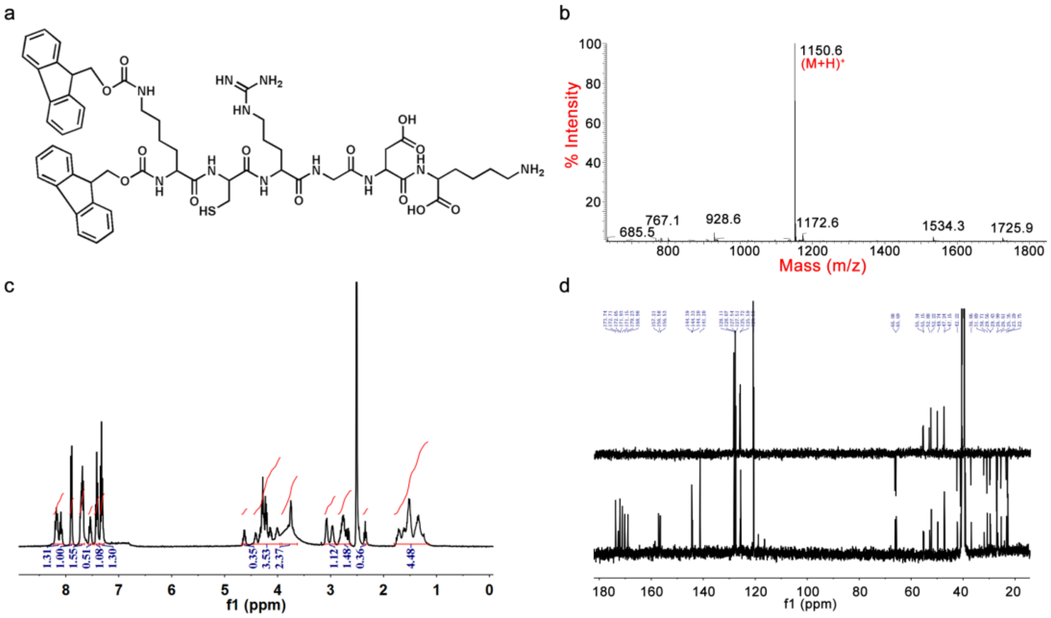

Supplementary Figure 2. Structural confirmation of Fmoc-KCRGDK (FK) peptide. (a) Chemical structure, (b) Mass spectra, (c) ^{1}H-NMR, and (d) ^{13}C-NMR of FK peptide.

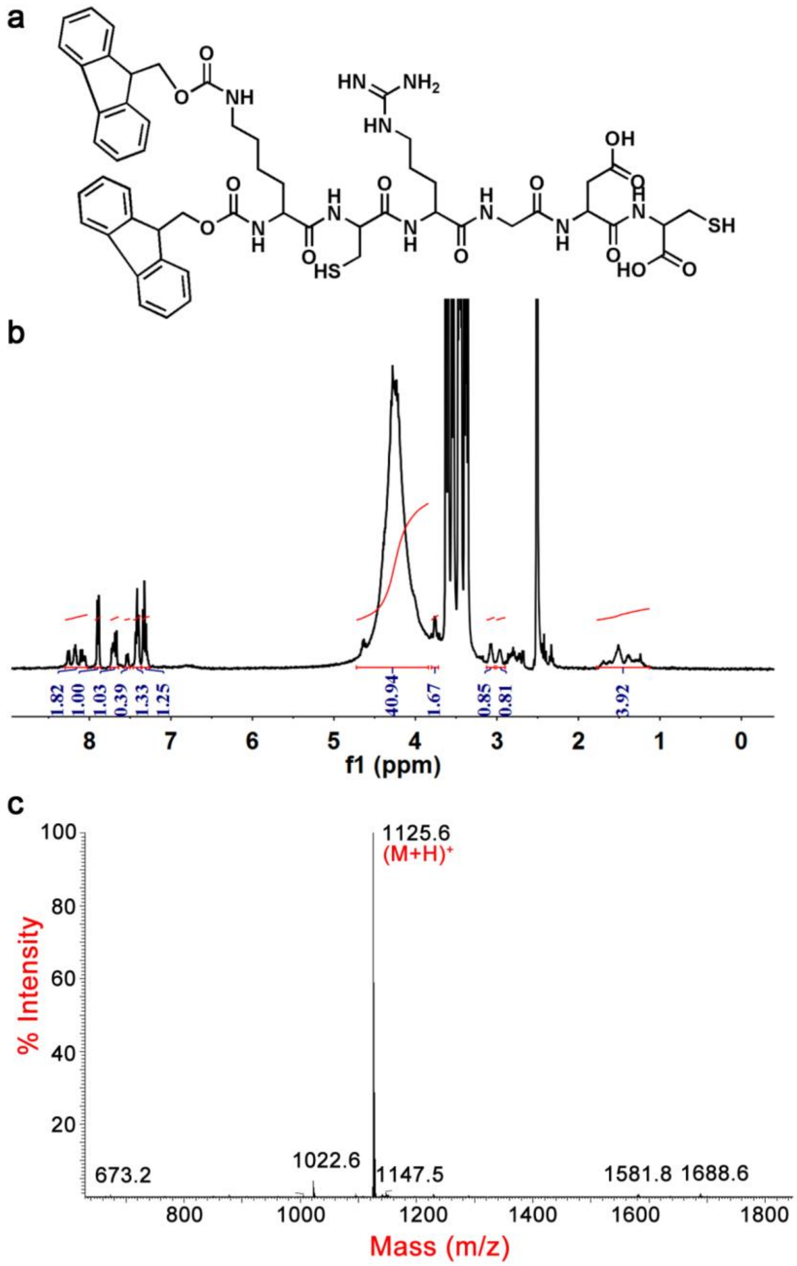

Supplementary Figure 3. Structural confirmation of Fmoc-KCRGDC (FC) peptide. (a) Chemical structure, (b) ^{1}H-NMR, and (c) Mass spectra of FC peptide.

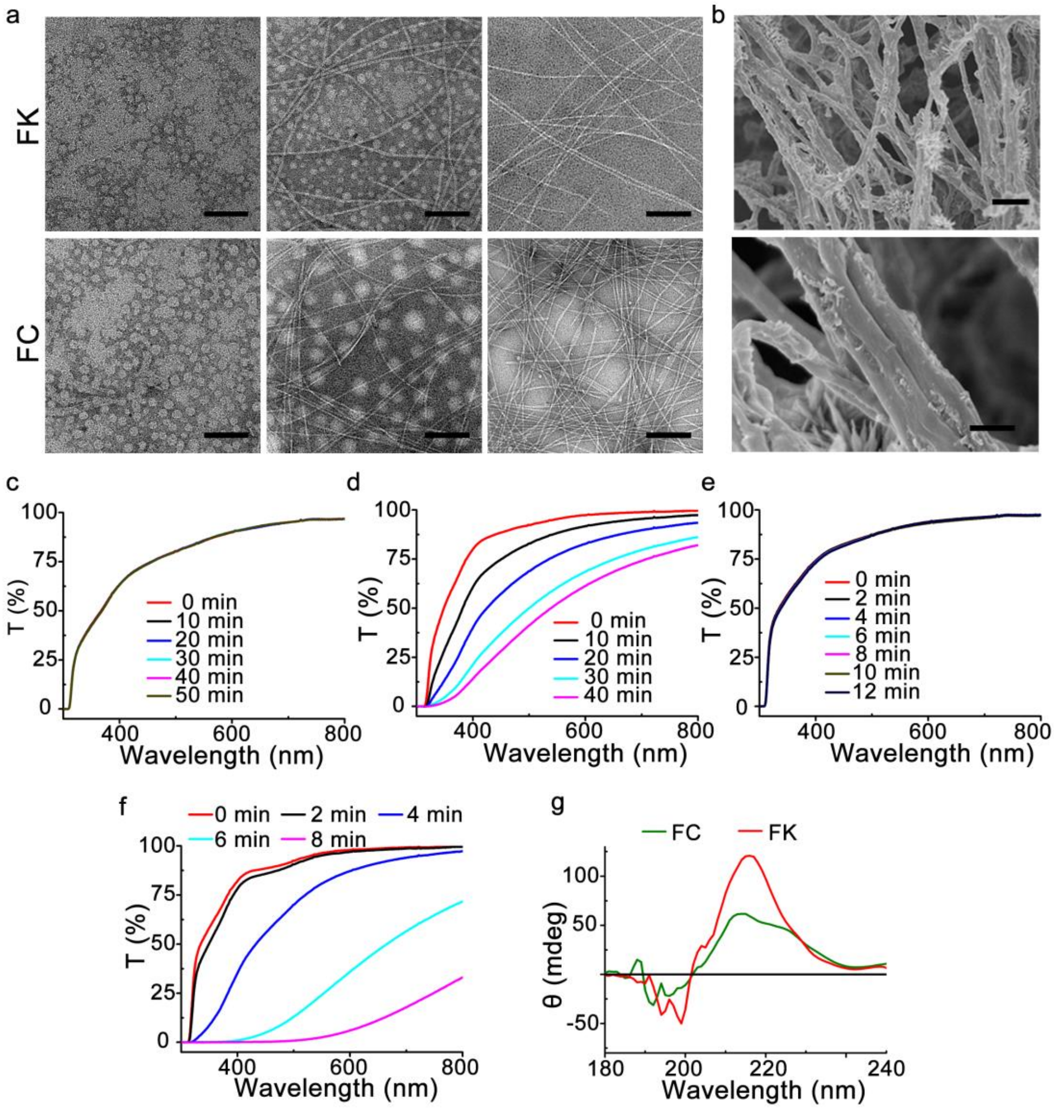

Supplementary Figure 4. Physical characterization of gelation process of the hydrogel matrix. (a) TEM images of FK and FC micelles and hydrogels examined before, under and after incubation at 70 ℃ (Scale bars, 50 nm). (b) SEM images of FK hydrogel (Scale bars, up: 2 μm; bottom: 500 nm). (c) Optical transmittance of FC hydrogel examined post the predetermined time durations at 25 or (d) 70 ℃. (e) Optical transmittance of FK examined post the predetermined time durations at 25 ℃ or (f) 70 ℃. (g) CD spectra of FK and FC hydrogels examined at a concentration of 0.5 mg/mL.

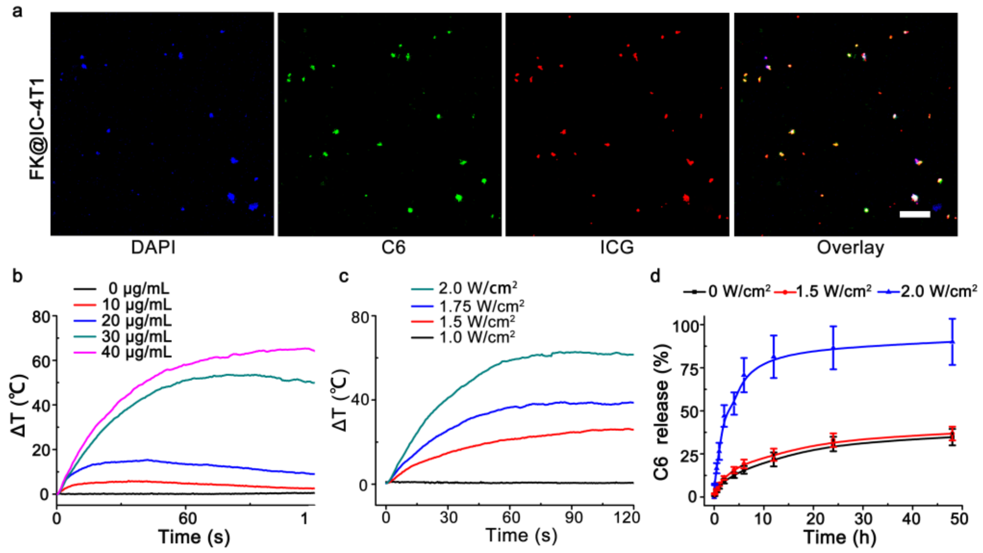

Supplementary Figure 5. Characterization of the tumor cell-loaded hydrogel matrix. (a) The confocal laser scanning microscopy images of FK hydrogels (0.5 % w/w) loaded with IC-4T1 (100 μL/2×10^6 cells, red: ICG, green: C6, blue: the nucleus) (Scale bar, 100 μm). Photothermal effects of FK@IQ-4T1 as a function of (b) ICG concentration (photo density: 2.0 W/cm^2), or (c) Photo density (ICG concentration: 30 μg/mL); (d) Laser-triggered C6 release from FK@IC-4T1. Data represent means ± s.d. (n=3).

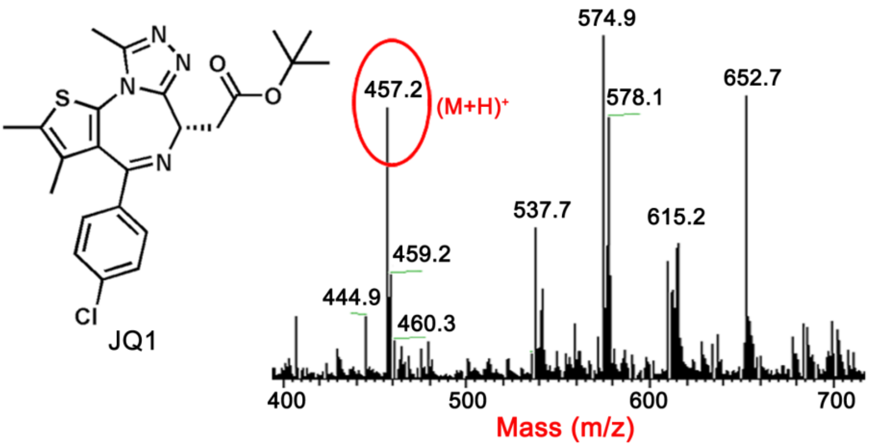

Supplementary Figure 6. Mass spectra of JQ1 released from FK@IQ-4T1 upon 808 nm laser irradiation.

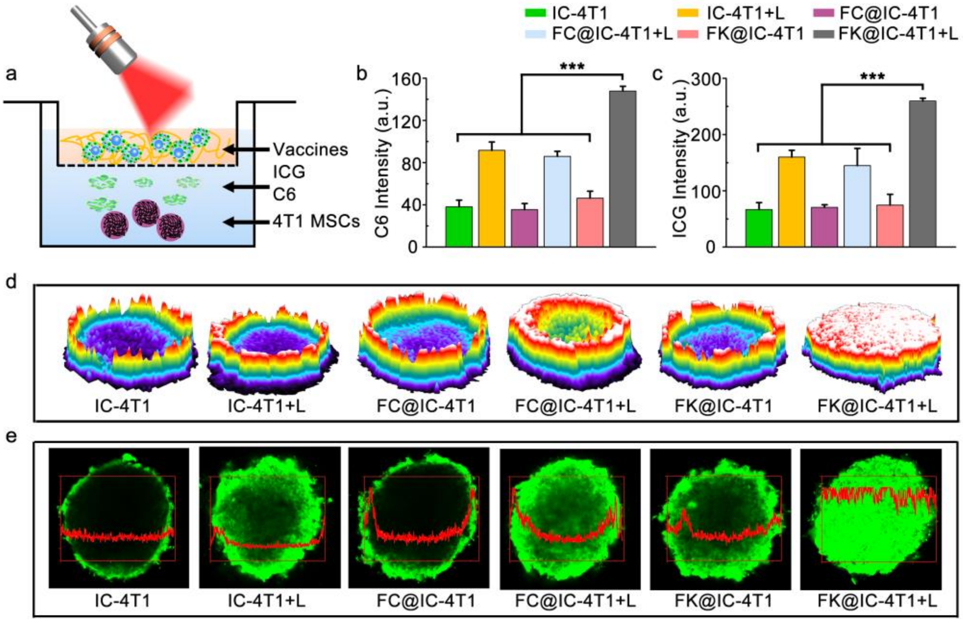

Supplementary Figure 7. Tumor penetrating capability of PVAX in 4T1 tumor cell-derived multicellular spheroid (MCS) model. (a) A schematic diagram illustrating the co-culture system with culture inserts. (b) Flow cytometry of intracellular accumulation of C6, and (c) ICG in 4T1 MCSs model. (d) 2.5-D CLSM images of FK@IC-4T1 distribution in 4T1 MCSs examined 12 h post vaccine incubation and laser irradiation. (e) CLSM images and fluorescence intensity profile of C6 examined at Z-axis of 150 μm. The significance of differences was evaluated by one-way ANOVA *** $p < 0.01$. Data represent means ± s.d. (n=3).

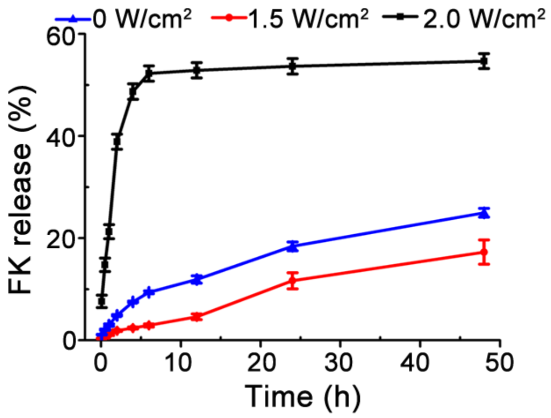

Supplementary Figure 8. Laser-triggered FK release profile from the hydrogel as a function of photodensity. Data represent means ± s.d. (n=3).

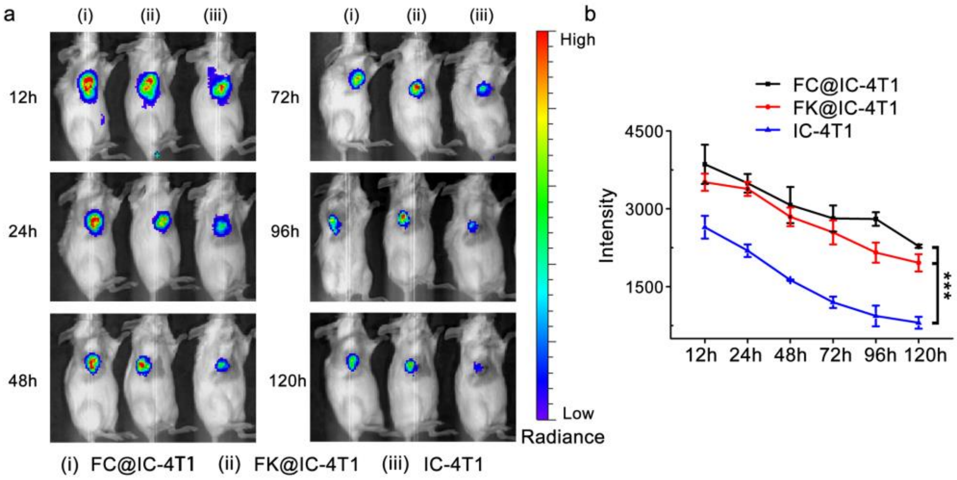

Supplementary Figure 9. Tumor accumulation and retention capability of PVAX *in vivo*. (a) Fluorescence examination of the intratumoral accumulation and retention of the intratumorally injected cancer vaccines *in vivo*. (b) Normalized ICG fluorescence intensity of intratumorally injected FK@IC-4T1 vaccine as a function of injection time. The fluorescence imaging *in vivo* was performed using ICG (Ex = 710 nm, Em = 780 nm). The significance of differences was analyzed by Unpaired student's *t*-test (two-tailed) *** $p < 0.01$. Data represent means ± s.d. (n=3).

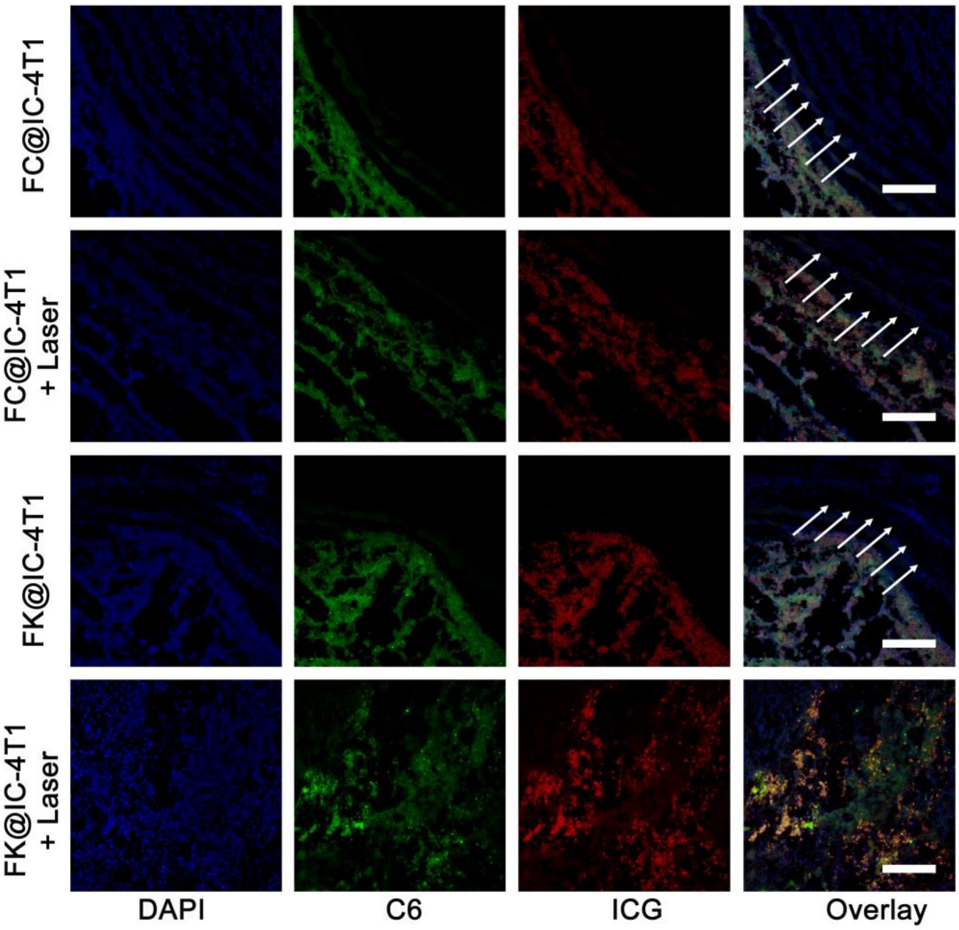

Supplementary Figure 10. CLSM examination of intratumoral penetration of ICG and C6 co-loaded FK or FC hydrogel in 4T1 tumor xenografts (the white arrows indicated the tumor burden restricting the penetration of vaccines (Scale bars = 400 μm).

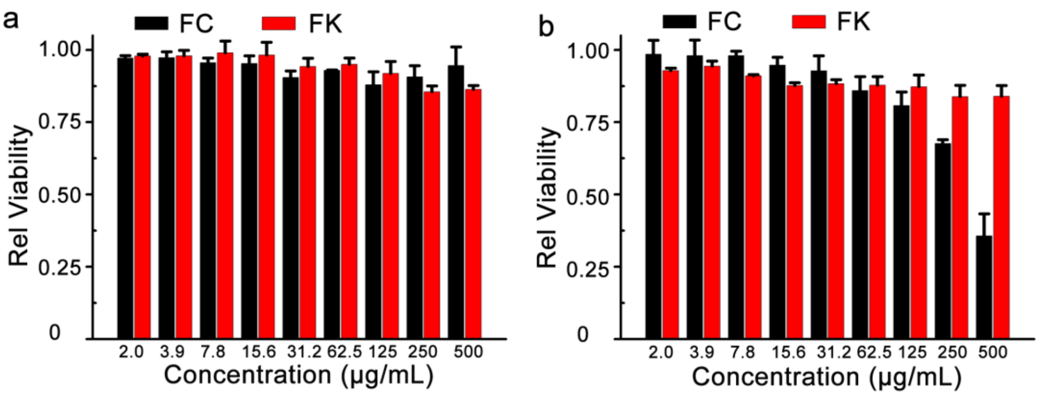

Supplementary Figure 11. Cytotoxicity assay of FK and FC hydrogels *in vitro*. The biocompatibility of FK and FC hydrogels in (a) RAW 264.7, and (b) BMDCs examined using SRB assay post 48 h incubation.

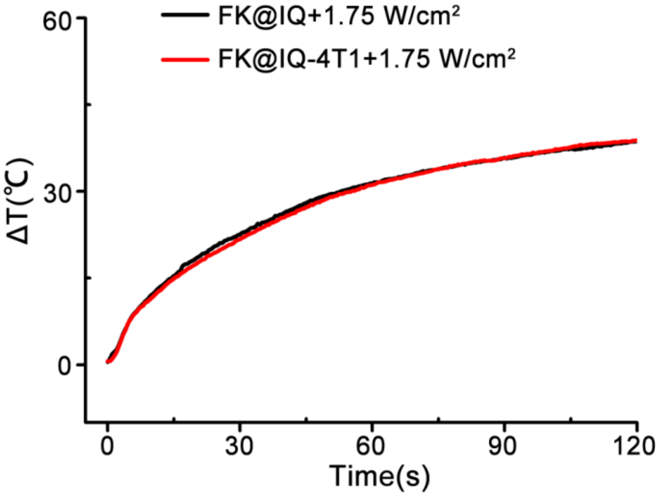

Supplementary Figure 12. FK-IQ and FK@IQ-4T1 induced comparable photothermal effects *in vitro* at an ICG concentration of 30 μg/mL upon 808 nm laser irradiation for 2 min.

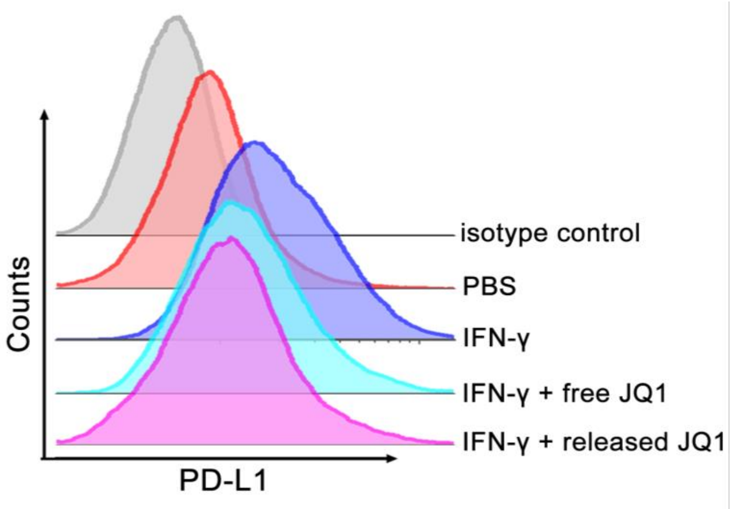

Supplementary Figure 13. Flow cytometric analysis of JQ1-induced downregulation of PD-L1 on the membrane of 4T1 cells (INF-γ: 20 nM; JQ1: 200 nM).

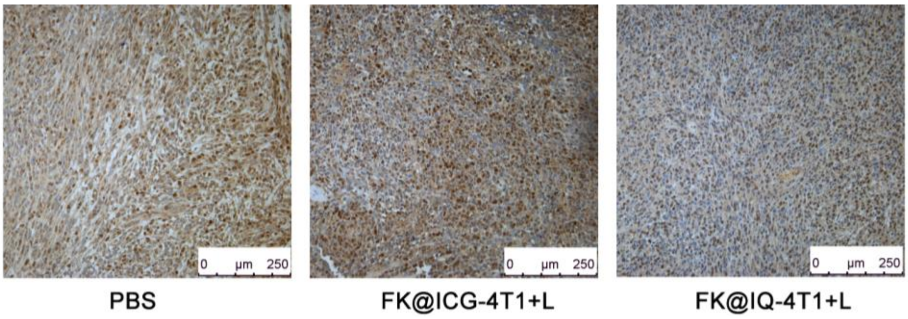

Supplementary Figure 14. Immunohistochemical assay of PD-L1 expression in 4T1 tumors examined at the end of the anti-recurrence study shown in Fig. 4a.

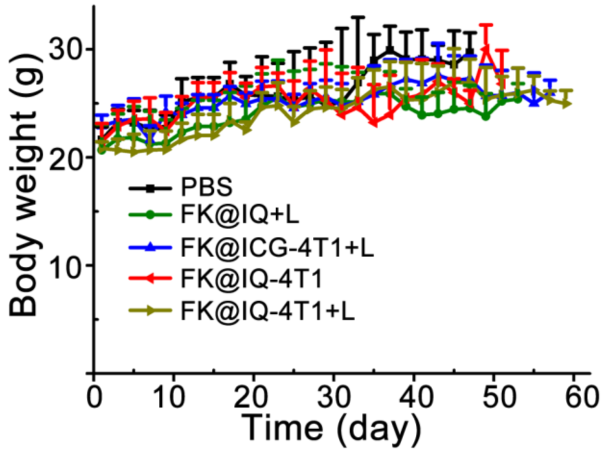

Supplementary Figure 15. Body weight change of the 4T1 tumor bearing mice recorded during the anti-recurrence study according to the treatment schedule shown in Fig. 4a.

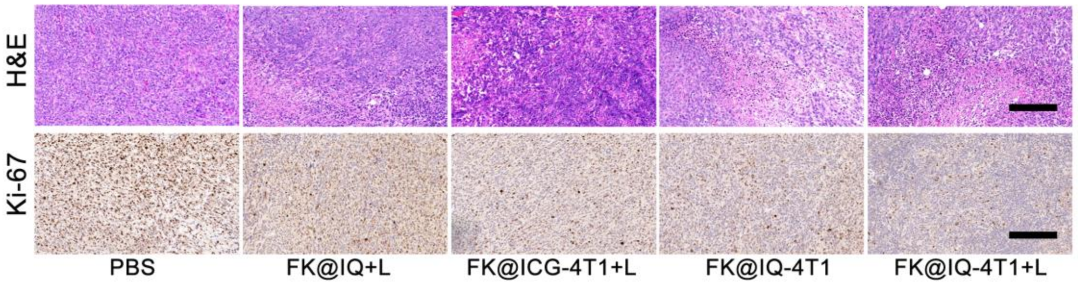

Supplementary Figure 16. H&E and Ki-67 staining of the 4T1 tumor sections at the end of anti-recurrence study using the autologous tumor model according to the treatment schedule shown in Fig. 4a (Scale bars = 200 μm).

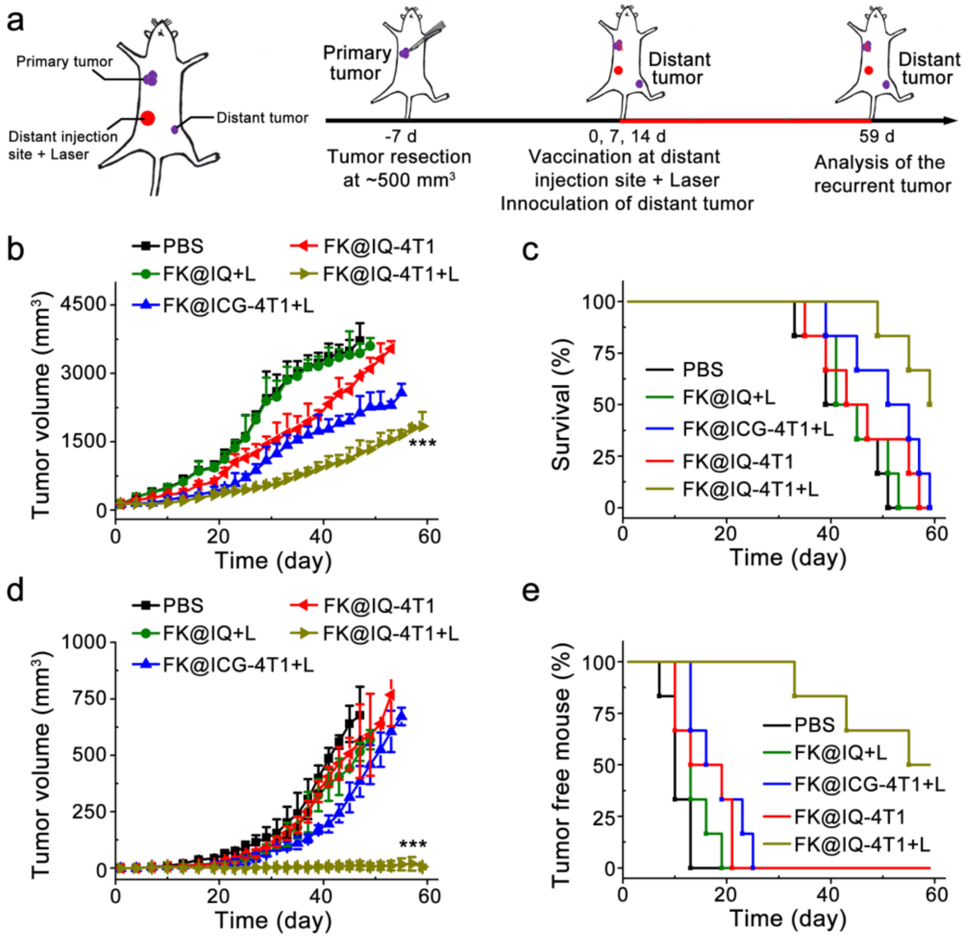

Supplementary Figure 17. **PVAX-performed immunotherapy for recurrent and metastatic 4T1 tumor xenografts *in vivo*.** (a) Treatment schedule for the immunotherapy; (b) Average tumor growth curves of the recurrent tumors receiving different treatments at the distant sites, laser irradiation for 2 min at photodensity of 2.0 W/cm^2. The mean tumor volumes were analyzed using one-way ANOVA. *** $p < 0.01$. Data represent means ± s.d. (n=6). (c) Survival percentage of the 4T1 tumor-bearing mice treated by surgery resection and PVAX-performed immunotherapy at the distant sites. (d) Average tumor growth curves of the distant tumors receiving different treatments at the distant sites, laser irradiation for 2 min at photodensity of 2.0 W/cm^2. The mean tumor volumes were analyzed using one-way ANOVA. *** $p < 0.01$. Data represent means ± s.d. (n=6). (e) Tumor-free percentages of the distant tumor rechallenged mice.

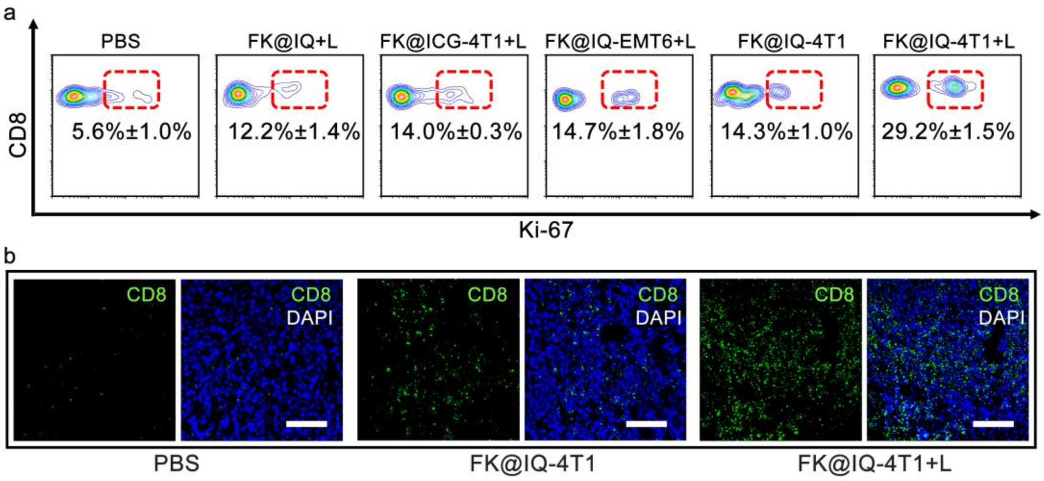

Supplementary Figure 18. T lymphocyte infiltration in the recurrent tumors treated according to the schedule showed in Fig 4a. (a) Proliferation activity of tumor infiltrating $CD8^+$ T cells in the recurrent 4T1 tumors after the indicated treatments. Data represent means ± s.d. (n=3). (b) Immunofluorescent examination of intratumoral infiltration of $CD8^+$ T cells in the recurrent 4T1 tumors examined at 3 days post the third treatment (Scale bars, 200 μm).

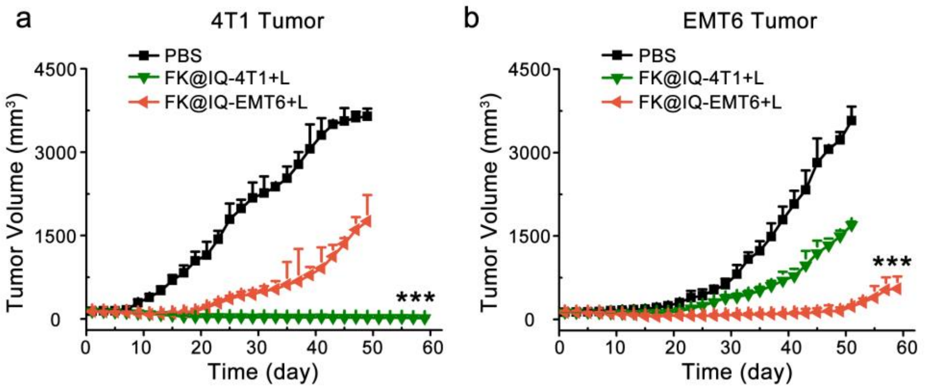

Supplementary Figure 19. PVAX-performed immunotherapy of the primary tumors according to the treatment schedule shown in Fig 4a. Tumor growth curves of (a) 4T1 and (b) EMT6 recurrent tumors upon different treatments (the treatment schedule was shown in Fig. 6a). The mean tumor volumes were analyzed using one-way ANOVA. *** $p < 0.01$. Data represent means ± s.d. (n=6).

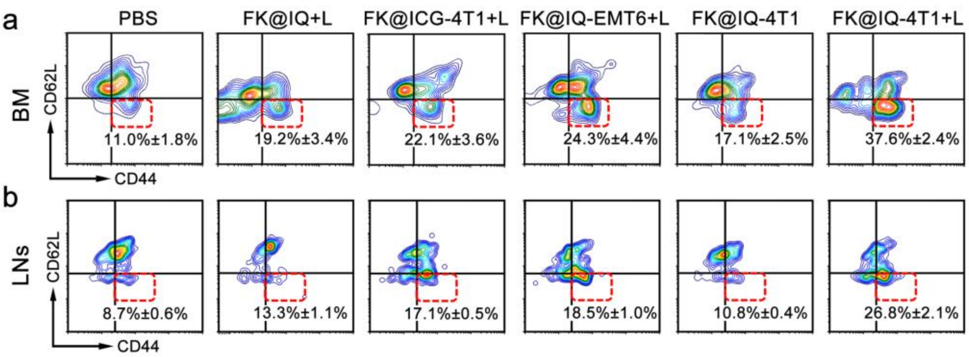

Supplementary Figure 20. **PVAX-induced long term immune memory effects.** Flow cytometry plots and proportions of effector memory T cells in the (a) bone marrow, and (b) the lymph nodes (gated on $CD8^+$ $CD11b^+$) examined at the same day for i.v. infusion of 4T1 cells. Data represent means ± s.d. (n=3).

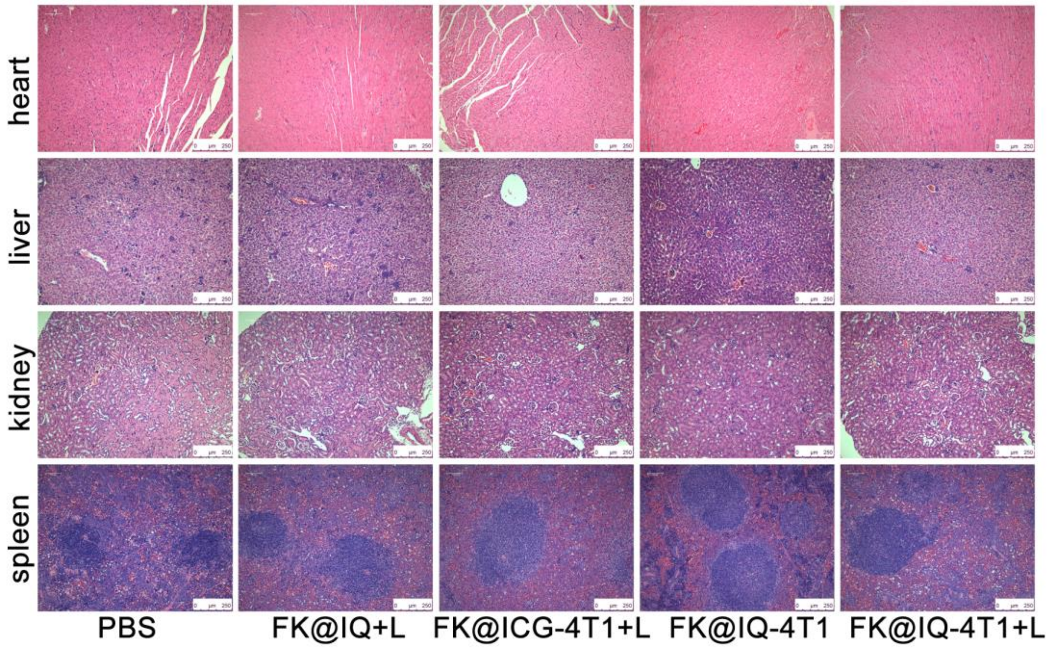

Supplementary Figure 21. H&E staining of the heart, liver, spleen and kidney of 4T1 tumor bearing mice examined at the end of anti-recurrence study using the autologous tumor model (The treatment schedule was shown in Fig. 7a, Scale bars = 200 μm).

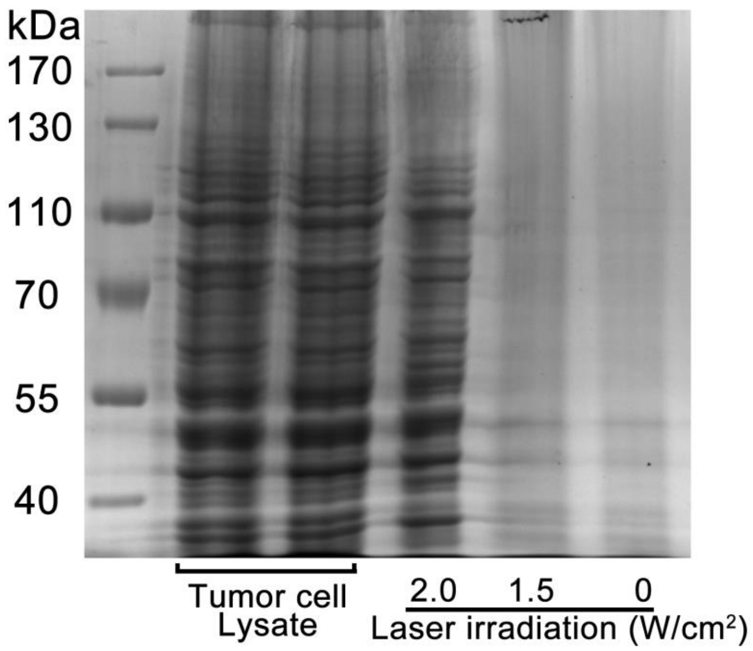

Supplementary Figure 22. The original SDS-PAGE gel for Fig.2f.
